# Theodicy perspective as an effect of the interpenetration of mental and religious issues during the COVID-19 pandemic in Poland

**DOI:** 10.1371/journal.pone.0355379

**Published:** 2026-09-02

**Authors:** Grzegorz Adamczyk, Arkadiusz Jabłoński, Piotr T. Nowakowski, Robert T. Ptaszek

**Affiliations:** 1 Institute of Sociology, Catholic University of Lublin, Lublin, Poland; 2 Institute of Pedagogy, Rzeszów University, Rzeszów, Poland; 3 Faculty of Psychology and Social-Political Sciences, Samarkand State University, Samarkand, Uzbekistan; 4 Institute of Philosophy, Catholic University of Lublin, Lublin, Poland; University of KwaZulu-Natal College of Health Sciences, SOUTH AFRICA

## Abstract

This paper aims to answer the question regarding the extent to which the various types of COVID-19 pandemic experiences differentiated the intensity of religious attitudes among Poles. For this purpose, the validity of a working hypothesis derived from Niklas Luhmann’s social systems theory was tested. According to this hypothesis, the interpenetration of mental and religious systems in times of a pandemic leads to the emergence of meanings that reorganise communication with other social systems. Thus, we sought empirical indicators of the strengthening of theodicy orientation within our respondents’ mental and religious systems in correlation with the pandemic situation. The data obtained from the statistically representative sample of 2,312 adults who self-identified as believers were processed using four measurement scales, and the working hypothesis was verified through stepwise linear regression analysis. The results obtained indicate that the COVID-19 experience was a dominant variable in shaping religious behaviour, to the extent that demographic factors under pandemic conditions turned out to be statistically insignificant. The observed interpenetration of mental and social systems went beyond these differences, as it concerned the very foundations of communication in an environment that introduced serious disruptions in the information flow between social subsystems and humans.

## Introduction

There is a saying in Polish: “When in fear, God is near.” It expresses the conviction that in the face of critical situations, humans seek help through divine intervention. This perfectly describes the tension between human expectations and the search for meaning in complex social systems. The paradox of coding and structuring communication in the social system is particularly evident during the COVID-19 pandemic. The system’s irritation caused by the virus is of particular interest, especially concerning religion. We will verify the validity of the hypothesis derived from Niklas Luhmann’s theory. According to this theory, the interpenetration of the mental and religious systems during a global crisis (e.g., a pandemic) prompts people to return to the basic question of theodicy, interpreting the pandemic, for example, as God’s punishment. This does not yet set a new programme for undertaking actions but is an attempt to organise communication with other social systems. Although many studies have examined religiosity during the COVID-19 pandemic, most sociological interpretations focus on changes in religious practices or beliefs rather than on their broader theoretical interpretation. This study applies a systems-theoretical perspective, viewing the pandemic as a disturbance that intensifies the interpenetration between mental and communication systems, thereby reframing the analysis of pandemic religiosity as a process of sense-making rather than merely a behavioural change.

### Theoretical Bases

Epidemics have always had significant economic and political repercussions, and people have often been led to believe they were living in the end times. Their reaction was characterised by apocalyptic fear, an attempt to read God’s message, or a search for the so-called scapegoats responsible for the situation [1,2]. The plague caused not only apocalyptic speculation, but also combinations of signs associated with it (wars, natural disasters). When an epidemic appeared, regardless of its other manifestations, the religious reaction was more reflective [3]. Christian churches, to redeem sins and obtain God’s mercy, called for common fasting and prayers, along with carrying relics in street processions, often accompanied by flagellants [2].

Medical developments at the end of the 19^th^ century made it possible to causally link microorganisms with infectious diseases, and epidemics as natural phenomena became the subject of science rather than eschatology [4]. However, the religious perspective has not ceased to be discussed and resurfaces repeatedly when we are faced with a new epidemic whose origin or course we do not fully understand [2]. This was the case with the Spanish flu in 1918 [5], AIDS at the end of the last century [6], and COVID-19 [7,8], which was also reflected upon and interpreted in apocalyptic terms [9].

Communication difficulties are one of the main social problems related to the COVID-19 pandemic that have affected the limits of meaning (selections of sense) in the operation of institutions (organisations) created to protect values considered essential from the point of view of humanity. Understanding how to link a specific social institution with the value system it was established to uphold is the subject of philosophical sociology [10]. In this regard, we will be particularly interested in the function of religion, which will be analysed through the approach initiated by Max Weber and further developed within the framework of Niklas Luhmann’s systems theory.

Weber used the sociological understanding of the phenomenon of religion to describe social phenomena during the Spanish flu epidemic. At that time, he noticed the regularity with which people become more religious when their lives and livelihoods are seriously threatened. In societies where religious beliefs are deeply rooted, the majority turn to religion for an explanation of the catastrophe that has struck their community [11]. Weber was the first to use Leibniz’s theodicy in sociological discourse research as a way to rationalise negative experiences, catastrophes, suffering, and pain. Theodicy is also at the centre of religious interpretations of reality in today’s pandemic crisis [12].

In sociology, a theodicy reference has been established as a set of meanings concerning God and his ability to interfere with the world, shaping individual consciousness and social communication. The misfortunes and disasters caused by things beyond human control have resulted in pointing to higher forces behind them. As researchers note, the theodicy perspective for explaining this phenomenon was of great importance in the history of mankind. Despite the announcement of God’s death, especially in the context of the Holocaust, the theodicy perspective still plays a significant role in interpreting human misfortunes [13,14].

Based on Luhmann’s systems theory, we will present such references as a conflict in the perspectives of observing meanings that update or negate the reality communicated within the religious subsystem and the objects of people’s conscious awareness during the COVID-19 pandemic. We want to go beyond using the conceptual categories of this theory to describe global phenomena regarding how each system has been affected by a pandemic and the disruptions it has caused in other systems [15]. This theory will also not be treated as a tool for making judgements about necessary systemic changes in post-pandemic times, without the ability to predict and explain current phenomena [16].

Luhmann’s theoretical perspective may provide a more adequate framework for studying communication disruptions between social subsystems and humans. In sociological research on the impact of a pandemic on human behaviour in a complex social system, people’s mental and physical abilities are activated by a system of interactions mediated by meanings [17]. The use of meanings connects and, at the same time, distinguishes the psychological system of consciousness and the social system of communication. From the point of view of mental experiences, communication can only take place consciously, but from the social perspective, communication is possible only as an event that transcends the unity of consciousness [18]. Therefore, it is necessary to look for theoretical reference points that enable empirical analysis of interactions, and at the same time, in the sense of the interpenetration model, may take into account the relationship between social action and the structural dimension [19].

The concept of interpenetration, meaning the interpenetration of the human (mental) system with the religious system, links the social system with human behaviour [20]. Humans as a psychic system constitute an environment for every such functional system for society, and vice versa.

Consciousness intervenes and thereby acquires the possibility of drawing boundaries for social systems precisely because these boundaries are not, at the same time, boundaries of consciousness. The same holds conversely: the boundaries of psychic systems fall within the communicative domain of social systems. In the course of orienting itself, communication is constantly forced to use what psychic systems have already assimilated in their consciousnesses and what they have not. This is possible because the boundaries of psychic systems are not also boundaries of communicative possibilities [20].

Thanks to experiencing and acting, humans are sensors providing religious organisations with information about the environment, maintaining higher complexity and less order, which implies greater freedom and unpredictability of behaviour. Society, for its part, can never be completely determined by human desires or expectations, and its complexity, in turn, always exceeds the understanding of individual mental systems [21]. The interpenetration of meanings between systems influences the observation of their particular elements. The contingency of what is observable as opposed to what is unobservable (developed into immanent or transcendent differences) determines the binary code of communication appropriate for the religious subsystem.

Self-referential autonomy at the level of individual societal subsystems was first established in the seventeenth and eighteenth centuries. Previously, the religious positioning of the world occupied this functional role. Perhaps one can say that the reference to God intended in all experience and action functioned as the secret self-reference of the societal system. One might have said that without God’s help no work could succeed. Societal as well as moral demands were fixed thereby. But the religious semantics was not formulated as society’s self-reference; it was (and still is) formulated as other-reference, as transcendence [20].

Every observable fact is accompanied by an unobservable transcendent correlate. Yet, this difference is itself observable in the form of meanings expressing the paradox and mystery of the sacred in contrast to the triviality of temporal objects [22]. Religion created a sublime communication system that, along with the pandemic, was subjected to irritation from its physical environment. This, in turn, revealed the paradox of its code, especially when juxtaposed with the code of the medical, legal, and political subsystems. Reducing the complexity of the religious system through to the code in force was enough to limit its contingency, regardless of changes in the environment [20]. In the era of uncertainty caused by the increasing complexity of the social system, many expectations structured by religious organisations have been irreversibly disrupted.

Communication using a religious code does not have a generalised symbolic medium. In modern society, religion has been reduced to one of society’s many functional systems. Its special function is inclusion, meaning the participation of excluded people in communication that constructs meaning (the difference between what is current and what is possible), which gives access to the transcendent world. “A God who experiences everything and is accessible through communication but who does not belong to society is a singular exception that exactly copies the recursive totality of the societal system itself, a duplication that makes it possible to experience the world in a religious way” [20].

It should be recognised that the theodicy code uses the code paradox (disease/health) from the medical (treatment) subsystem, where health is unobservable. As the absence of disease, health can be understood as both absence and unobservability associated with the highest unobservability, that is, God. The structure of mental systems giving meaning to social processes and the actions that result from them is always in the religious sphere and is irritated by disease, and therefore by some form of moral evil. This is especially severe for the system when it is associated with the prohibition of going to church and participating in religious services. Such constraints, necessarily included in religious communication, in different ways fit the formed self-organisation of a variety of interactions [23]. Thanks to such interaction, people are influenced by the partial systems that make up the social system of politics, state, media, and health service. However, unlike symbolic interactionism, the social dimension is not reduced here to a simple interaction between individuals, but leaving people (socially identified assemblages of expectations) in the interaction process guides the selection of perceptions and outlines the perspectives of social significance.

Depersonalisation of the view of society in systems theory during a pandemic has shown its power to adhere to the perceptions of many religious people about the contemporary reality of pandemic times. They see the political, legal, medical, media, and economic subsystems as creating an environment that disturbs humans’ possibility to fulfil their functions [24]. This is how they interpret any restrictions imposed on people as motivated by the need to protect the efficiency of the medical care system. In this context, the good of humans could at best be understood systemically as a proposition of meaning accepted by the majority of public opinion, or at least by opinion leaders, which is an integral part of the political system.

### Research goals

Referring to Weber’s [25] previously mentioned classic thesis about the negative correlation between physical/economic security and religiosity, we would like to answer the following question: to what extent do the different types of experiences related to the COVID-19 pandemic, conceived as codes from the medical subsystem, differentiate the level of intensity of Poles’ religious attitudes? We will measure this based on respondents’ declarations describing the importance of religion in their life, expressing their religious experiences. In essence, persons practicing institutionalized religiosity may discover the meaning of life, feel closeness to the ultimate reality, and build the awareness that there is salvation in God [26]. Religiosity outside the church can play a similar role. A sense of connection with a supernatural force can serve a sense-making function for everyday life, even if this connection is not legitimized by membership in an institutional church [27]. From this point of view, it can be expected that the binary meaning code for health/disease from the medical subsystem, different for other types of COVID-19 experiences, will coexist with different types of religious experience expressed by the self-declaration of the intensity of one’s religious attitude.

This experience, however, is not the same as accepting the assumptions of particular churches but is a return to the source and a non-confessional reference to divine transcendence. According to the assumptions of Luhmann’s systems theory, such a semantic return to God as a natural point of reference for finding meaning in life is almost absent in human experiences today. Therefore, it can be assumed that the theodicy orientation will be strongest among people who, on the one hand, declare the highest level of intensity of religious attitudes (religious experience) and, on the other hand, seek religious meaning in the COVID-19 pandemic. Hence, we investigate the extent to which the COVID-19 pandemic is given a religious meaning, as well as the degree to which different types of religious experience are linked to different perceptions of the COVID-19 pandemic in the theodicy sense. ”The centrality of intensive interactive rituals for producing the communal benefits of religion (e.g., social support, emotional catharsis, perceived healing) ensures that there will be persistent tension between many religious groups’ desire for in-person gatherings and the social distancing requirements necessary to limit the spread of COVID-19” [28]. In this way, we will search for empirical indicators of the effect of the interpenetration of the mental and religious systems that result in the strengthening of the theodicy orientation.

The pandemic is a unique experience due to the new conditions of human interpenetration with both the political subsystem (the organisation of public safety) and the religious subsystem (the organisation of religious practices). This may result in the coexistence of different meanings depending on the types of religious experience (measured by the intensity of religious attitudes) and different levels of acceptance of the sanitary restrictions in places of religious worship. Therefore, we will be looking for answers to the following questions: to what extent do believers in Poland accept the equivalence of the rules of the sanitary restrictions in places of religious worship and in other public places, and whether different degrees of acceptance of the sanitary restrictions coexist with different levels of the self-declaration of religious faith.

We expect that the specific nature of the interpenetration of the mental and religious systems during the COVID-19 pandemic is linked to a certain type of relationship between religious experience (in terms of the intensity of religious attitude) and the type of COVID-19 disease experience, the scope of the pandemic’s perception in the religious sense, and the degree of acceptance of sanitary restrictions in places of worship. Since this parameter of religious experience is closely related to other parameters of religiosity, including the ritualistic parameter [26], we expect that the scope of religious practices will significantly differentiate the above-mentioned relationships. We also expect that the relationships between religious experience and other variables will vary by gender and age groups.

A systematic examination of the above differentiations will allow us to verify the H1 research hypothesis, which assumes that the self-declaration of religious faith (religious experience index) is stronger with more severe experiences of the disease caused by COVID-19 (mental system) when the pandemic is given a religious meaning (theodicy orientation). This effect should be stronger among regular practitioners than non-practitioners or irregular practitioners, older rather than younger individuals, women rather than men, and opponents of sanitary restrictions in places of religious worship than among its supporters. If the directions of association contained in this hypothesis are confirmed, we will come closer to answering the main research question of whether the interpenetration of the mental and religious systems in times of a pandemic may increase the tendency towards a theodicy orientation, especially in specific segments of the population (regular practitioners, older individuals, women, and opponents of sanitary restrictions in places of religious worship).

## Materials and methods

### Research sample

The research was carried out in July 2021 using an online survey completed by 3,032 participants from a panel of Internet users from Ariadna Research Company. The decision regarding the sample size was determined by two assumptions. Firstly, the acceptable statistical error should not exceed approximately 2 percentage points, which requires a sample of about 2,500 respondents in relation to the 2021 general population of 31.2 million adult inhabitants of Poland. Secondly, the hypotheses tested in this study required respondents who declared themselves to be either regularly, irregularly, or non-practising believers. Thus, we limited our analysis to 2,312 respondents who self-declared a religious faith regardless of whether they practised religion or not. The remaining part of the sample of 720 respondents who declared themselves as non-believers was not included in the analyses.

Once the survey had been approved by the Ethical Committee of the Institute of Sociological Sciences at the Catholic University of Lublin (decision 14/DKE/NS/2021), the respondents’ recruitment for the study was carried out on July 6–8, 2021. Participant consent was obtained by having respondents personally tick the appropriate box in the online questionnaire. Participation in the surveys was fully anonymous. Because the fieldwork was carried out by an external company, the authors of the manuscript did not have access to any information that could identify the survey participants.

The initial sample of the entire population aged 18 and over as well as the target sample of believers aged 18 and over used in the study are statistically representative of the adult population of Poland based on four key sociodemographic variables: age, gender, size of place of residence, and region (voivodeship) of residence. The distribution of the variables in the sample differs by a maximum of about 2–3 percentage points from the results for the general population (Table 1). As these differences do not exceed the statistical margin of about 2–3%, it can be concluded that the sample is well aligned with the sociodemographic structure of the general population.

The structure of the research samples used in the analyses presented below is as follows.

**Table 1:** Socio-demographic structure of the study sample.

	The sample of the population 18+		The sample of believers 18+		Population of Poland aged 18+ *, *N = 31,214,992*
	*N=*	*%*	*N =*	%	
**Gender**					
Men	*1,446*	47.7	*1,052*	45.5	47.6
Women	*1,586*	52.3	*1,259*	54.5	52.4
**Age**					
18–24	*260*	8.6	*180*	7.8	8.5
25–34	*506*	16.7	*387*	16.7	16.4
35–44	*610*	20.1	*467*	20.2	20.2
45–54	*482*	15.9	*378*	16.4	16.2
55 and over	*1,174*	38.7	*899*	38.9	38.8
Confession **					
Roman Catholic	*2,431*	80.2	*2,165*	93.7	89.8
Orthodox	*46*	1.5	*41*	1.8	0.5
Protestant	*28*	0.9	*24*	1.1	0.3
Other	*37*	1.2	*29*	1.3	0.8
No confession	*490*	16.2	*52*	2.2	8.6
**Declaration of Faith And Religious Practices**					
Believer and regular practitioner	*680*	22.4	*680*	29.4	-
Believer and irregular practitioner	*923*	30.3	*923*	39.9	-
Believer and non-practising	*709*	23.4	*709*	30.7	-
Non-believers	*720*	23.9	*-*	-	-
**Education**					
Elementary/lower secondary school	*-*	-	*53*	2.3	-
Basic vocational	*-*	-	*255*	11.1	-
High school	*-*	-	*999*	43.2	-
Higher education	*-*	-	*1,005*	43.5	-
**Region (Voivodeship)**					
Dolnośląskie	*230*	7.6	*164*	7.1	7.6
Kujawsko-Pomorskie	*148*	4.9	*112*	4.8	5.4
Lubelskie	*168*	5.5	*134*	5.8	5.5
Lubuskie	*59*	2.0	*39*	1.7	2.6
Łódzkie	*217*	7.2	*169*	7.3	6.4
Małopolskie	*253*	8.3	*198*	8.6	8.8
Mazowieckie	*517*	17.0	*373*	16.1	14.0
Opolskie	*64*	2.1	*50*	2.1	2.6
Podkarpackie	*156*	5.2	*138*	6.0	5.5
Podlaskie	*113*	3.7	*91*	4.0	3.1
Pomorskie	*144*	4.8	*97*	4.2	6.0
Śląskie	*319*	10.5	*234*	10.1	11.8
Świętokrzyskie	*107*	3.5	*80*	3.4	3.3
Warmińsko-Mazurskie	*105*	3.5	*82*	3.5	3.7
Wielkopolskie	*311*	10.3	*262*	11.3	9.0
Zachodniopomorskie	*121*	4.0	*88*	3.8	4.5
**Town Size And Class**					
Village	*1,197*	39.5	*966*	41.8	39.5
Small town (fewer than 20,000 inhabitants)	*378*	12.5	*296*	12.8	13.3
Medium-sized city (from 20,000 to 99,000 inhabitants)	*590*	19.5	*452*	19.6	19.3
Large city (from 100,000 to 500,000 inhabitants)	*486*	16.0	*344*	14.9	16.2
Big city (over 500,000 inhabitants)	*381*	12.6	*252*	10.9	11.7

*Source: Authors’ research*

* According to the data of the Main Statistical Office of Poland

** According to the results of the 2021 National Census (Base: Persons who did not refuse the response to the question about the confession; 79.5% of the participants of the National Census)

### Measurement tool

The questionnaire used in the study consisted of 35 closed questions in the main part and 9 specific questions. In the main part, only closed questions (30) and semi-open questions (5) were used. Most of the questions were answered with a single response by the respondents using a five- or seven-point Likert scale (21). The remaining questions were either multiple-choice or single-choice options on a shorter numerical scale or dichotomous.

To verify the H1 hypothesis, the measurement results were obtained based on four measurement scales (self-declaration of religious faith, COVID-19 disease experience, religious meaning of the pandemic, and sanitary restrictions), and two demographic questions (gender, age) were used. The Self-Declaration of Religious Faith Scale was created using four questionnaires describing the respondent’s assessment: the importance of religion in one’s life (P1), the impact of the pandemic on one’s religious faith (P3), the impact of the pandemic on one’s relationship with God (P4), and one’s assessment of the pandemic as a motivation for greater religious involvement (P5). First, the results obtained from the above questions were recorded to produce equal-length five-degree variables with the same direction of measurement (with a midpoint signifying indecision on the topic). The variables prepared in this way were subjected to factor analysis using the principal components method with varimax rotation and then to reliability analysis. The results of both analyses clearly indicate that the above variables can be used to construct the Self-Declaration of Religious Faith Scale (all variables form a common component in the factor analysis; Cronbach’s alpha = 0.762). The scale ranges from 4 to 20, while maintaining properties of a normal distribution (skewness = 0.411; kurtosis = 0.751).

The COVID-19 Disease Experience Scale was created using the indexing technique based on a question describing one’s experience with COVID-19: ranging from personal experience due to infection and hospitalisation; through experiencing the infection, hospitalisation, and death of a family member; to experiencing the infection, hospitalisation, and death of a close friend. The indexation procedure was based on the following scheme: the respondent obtained the appropriate number of points depending on the situation they experienced during the epidemic: the death of a family member as a result of COVID-19 disease – 4 points; the death of a close friend as a result of COVID-19 disease – 3 points; personal hospitalisation due to COVID-19 disease – 4 points; hospitalisation of a family member due to COVID-19 disease – 3 points; hospitalisation of a close friend due to COVID-19 disease – 2 points; one’s personal experience of COVID-19 disease – 3 points; COVID-19 disease of a family member – 2 points; and COVID-19 disease of a close friend – 1 point. Using the above indexing scheme led to the creation of a scale ranging from 0 to 22. Subsequent recoding to an eight-point scale produced a distribution close to normal (skewness = 0.332; kurtosis = −0.849).

The Religious Meaning of the Pandemic Scale was created based on survey items describing how the respondent interprets the pandemic’s meaning in light of religious beliefs (P12) and the role of the pandemic in increasing one’s religious involvement (P5). In the first step, an indexation procedure based on P12 was carried out. For this question, the respondent could select up to three answers from a pool of ten options, each referring to different ways of interpreting the pandemic as an event of religious significance. The respondent obtained one point for each selected answer. In the second step, the above indexation was combined with the results of responses to the question describing the extent to which the respondent viewed the pandemic as a motivation for increasing religious commitment.

As a consequence, every respondent was rated on a scale of 1–8, where 1 indicates no selection of any answer suggesting a religious interpretation of the pandemic, along with a simultaneous declaration that the pandemic was not a motivator for increased religious involvement at all. On the other hand, 8 represents the maximum range of interpreting the pandemic as an event with religious significance (three selected responses) while declaring that the pandemic was a strong motive for increased religious commitment. The distribution of results on such a scale is close to normal (skewness = 0.443; kurtosis = −0.428).

In turn, the Sanitary Restrictions Scale was created using three survey items describing a respondent’s opinion on the autonomy of churches or religious associations in relation to public authorities regarding the principles of restrictions on the exercise of religious worship during the pandemic (P25), on the proper functioning of a church during the pandemic (P28), and on the validity of the premise for closing churches to the faithful in a situation of more than 30,000 daily infections (P34). Initially, the results obtained from the above questions were recoded to give equal-length five-point variables with the same measurement direction (with the midpoint indicating no opinion on the topic). The variables prepared in this way were subjected to factor analysis using the principal components method with varimax rotation, followed by a reliability analysis. The results of both analyses clearly show that the above variables can be used to construct the Sanitary Restrictions Scale (all variables form a common component in factor analysis; Cronbach’s alpha = 0.615). The scale ranges from 3 to 15, while maintaining the properties of a normal distribution (skewness = −0.134; kurtosis = −0.319).

### Data analysis

Data analysis verified that the H1 hypothesis was carried out separately for three categories of respondents (research subsamples): believers and regular practitioners (N = 680), believers and irregular practitioners (N = 923), and non-practising believers (N = 709). All the described procedures of data transformation and analysis (recoding, creating new variables, indexation, factor analysis, and Cronbach’s alpha reliability analysis) were performed using IBM SPSS software.

## Results

Based on the interpretation of the results according to the scales proposed by Faber and O’Guinn [29,30], five groups of respondents can be distinguished according to their self-declaration of religious faith, assessed in the context of the potential impact of the COVID-19 pandemic (Table 2). The first group achieved a score on the Self-Declaration of Religious Faith Scale of at least two standard deviations above the mean and includes 5% of respondents. These individuals report a very significant strengthening of their religious faith experience due to the pandemic’s influence (mean value = 12.69; standard deviation = 2.82). The second group consists of respondents who rate the pandemic’s impact as enhancing their religious faith experience slightly less than the representatives of the first group, although these ratings remain high compared to the entire population of believers (between one and two standard deviations above the mean). Eight percent of the respondents belong to this group. The third group comprises individuals who evaluate the impact of the pandemic as strengthening their religious faith experience significantly less than those in the first and second groups. Nevertheless, these ratings are still above average compared to the entire population of believers. This group includes 40% of respondents who rank between the mean and one standard deviation above the mean on the Self-Declaration of Religious Faith Scale. The group of individuals who are unaware of the pandemic’s impact on their religious faith experience includes 35% of respondents, with scores between the mean and one standard deviation below the mean on the self-declaration scale. The remaining respondents (12%) should be considered a group of individuals who do not perceive any change in their level of religious faith experience during the COVID-19 pandemic. The number of individuals in each group varies depending on the extent of their religious practice.

**Table 2:** Results on the Self-Declaration of Religious Faith Scale by scope of religious practices.

	Entire population of believers	Regularly practising believers	Irregularly practising believers	Non-practising believers
*N =*	*2,312*	*680*	*923*	*709*
Mean value	12.69	14.75	12.73	10.65
Standard deviation	2.83	2.68	2.15	2.21
Group 1	5%	9%	3%	2%
Group 2	8%	11%	10%	13%
Group 3	40%	22%	47%	37%
Group 4	35%	52%	28%	34%
Group 5	12%	6%	12%	14%

*Source: Authors’ research*

While 20% of regular practitioners claim that the pandemic had a significant impact on their religious faith experience (groups 1 + 2), this proportion is 13% for irregular practitioners and 15% for non-practising believers. At the same time, the lowest percentage of individuals denying any impact of the pandemic on their religious faith experience is observed among regular practitioners, while this rate among irregular practicioners and non-practising believers is is approximately twice as high.

The respondents report a varied range of experiences concerning the consequences of the COVID-19 pandemic. Nearly one-third of the respondents (31%) had no experience with COVID-19, meaning that they themselves, or any of their family members or close friends, had not been affected. Nearly 16% of the respondents were infected with the coronavirus, including over 1% who were hospitalised for this reason. About one-third of the respondents (32%) reported that someone in their family had been infected with the coronavirus, including 7% who confirmed that a family member had been hospitalised due to the infection. Nearly 6% of the respondents reported that a family member had died from COVID-19. Also, about one-third of the respondents (32%) indicated that one of their close friends was infected with the coronavirus, including 13% who reported that this person had been hospitalised. Moreover, one-tenth of the respondents experienced the death of a close friend due to COVID-19. To sum up, 43% of the respondents had an above-average experience of COVID-19, as indicated by scores above the mean on the COVID-19 Disease Experience Scale.

Almost every fifth respondent (18%) agreed, to a greater or lesser extent, with the statement that “the COVID-19 pandemic has given a new meaning to their religious commitment.” The opposite opinion was expressed by a twice as high percentage of respondents (37%), and the remaining 45% of the respondents either stated that there was no change in their religious commitment due to the pandemic or were unable to take a position. Overall, nearly half of the respondents attributed no religious significance to the pandemic (46%). On the other hand, those who perceive a certain religious meaning in the pandemic most often interpret it as a chance given by God to reflect on their lives (22%), as a test of faith (17%), and as God’s call to reflection (17%). Taking into account the results on the Religious Meaning of the Pandemic Scale, it can be concluded that nearly half of Poles (47%) who identify with religious faith (76% of the entire population) assign the pandemic a more or less religious meaning (i.e., results above the mean on the scale).

The results describing the respondents’ attitudes toward the sanitary restrictions show a clear division into two groups of relatively similar size. The first group believes that, during the pandemic, churches should remain open and conduct religious services as they did before the pandemic or with only certain restrictions (50%). The second group favours significantly limiting the functioning of churches (reserved only for special services or personal prayers), or even their complete closure (39%), or have no opinion on this matter (16%). Support for limiting the functioning of churches increases with the daily number of infections: 61% of the respondents support closing churches to the faithful, to a greater or lesser extent, when the daily number of infections exceeds 30,000.

Twenty-two percent of the respondents hold the opposite opinion, and 17% were unable to take a position in this respect. In addition, half of the respondents, to a greater or lesser extent, give priority to decisions made by public authorities regarding the limitation of church activities, while some respondents do not see any need to agree with churches or religious associations in this regard (26%). Slightly more than a quarter of the respondents (28%) take a separate position, believing that every church or religious association should decide for itself, at its discretion, about restrictions on religious worship services during the pandemic (10%), or within a framework generally determined by public authorities (18%). The remaining 22% of the respondents do not have an opinion on this issue.

The H1 hypothesis was verified based on the results of the stepwise linear regression analysis. The predictors were included in the model according to five steps, from the COVID-19 Disease Experience Scale (step 1); through the Religious Meaning of the Pandemic Scale (step 2), age (step 3), gender (step 4); and finally the Sanitary Restrictions Scale (step 5). The analysis was performed separately for three subsamples: persons declaring themselves as regular practicioners, persons declaring themselves as irregular practioners, and persons declaring themselves as non-practising believers.

In the case of the first category (Table 3), i.e., regularly practising believers, a slightly weaker self-declaration of religious faith coexisted with a more severe experience of the disease caused by COVID-19. The level of self-declaration of religious faith decreases by 0.186 points on a 17-point scale (± 0.051 points) along with an increase in the score on the COVID-19 Disease Experience Scale by one point (on an eight-point scale). The opposite effect is observed in the case of the Religious Meaning of the Pandemic Scale. Here, the rule is that the more religious meaning assigned to the pandemic, the higher the self-declaration of religious faith. An increase of one point on the eight-point Religious Meaning of the Pandemic Scale coincides with an increase in the level of self-declaration of religious faith by 0.945 points (± 0.045 points).

**Table 3:** Results of the stepwise linear regression analysis for the subsample of regular practitioners.

	B	Std. error	ß	T	*p*	Collinearity	
						Tol.	VIF
Step 1							
Constant	14.821	0.280		52.918	< 0.001		
Illness experience	−0.112	0.070	−0.073	−1.593	0.112	1.000	1.000
Step 2							
Constant	10.977	0.275		39.941	< 0.001		
Illness experience	−0.195	0.051	−0.127	−3.813	< 0.001	0.994	1.006
Pandemic’s religious significance	0.948	0.046	0.689	20.773	< 0.001	0.994	1.006
Step 3							
Constant	10.093	0.359		28.103	< 0.001		
Illness experience	−0.190	0.051	−0.123	−3.756	< 0.001	0.993	1.007
Pandemic’s religious significance	0.945	0.045	0.687	20.992	< 0.001	0.994	1.006
Age	0.018	0.005	0.123	3.752	< 0.001	0.999	1.001
Step 4							
Constant	10.120	0.430		23.545	< 0.001		
Illness experience	−0.189	0.051	−0.123	−3.729	< 0.001	0.986	1.014
Pandemic’s religious significance	0.945	0.045	0.687	20.969	< 0.001	0.993	1.007
Age	0.018	0.005	0.122	3.727	< 0.001	0.993	1.007
Gender	−0.018	0.159	−0.004	−0.113	0.910	0.986	1.014
Step 5							
Constant	10.260	0.511		20.090	< 0.001		
Illness experience	−0.186	0.051	−0.121	−3.641	< 0.001	0.973	1.028
Pandemic’s religious significance	0.945	0.045	0.687	20.943	< 0.001	0.993	1.007
Age	0.018	0.005	0.121	3.674	< 0.001	0.987	1.013
Gender	−0.022	0.160	−0.005	−0.139	0.890	0.983	1.017
Sanitary restrictions	−0.016	0.031	−0.017	−0.510	0.610	0.979	1.021

*Source: Authors’ research*; Step 1: ΔR² = .003, *p* = .112; Std. error = 2.41557; Step 2: ΔR² = .475, *p* < .001; Std. error = 1.75266; Step 3: ΔR² = .489, *p* < .001; Std. error = 1.72914; Step 4: ΔR² = .488, *p* < .001; Std. error = 1.73093; Step 5: ΔR² = .487, *p* < .001; Std. error = 1.73228.

Interestingly, assigning a religious meaning to the pandemic does not “abolish” the effect of severe illness coexisting with a reduced level of self-declaration of religious faith. This effect persists even after including other variables in the model. Among these, statistically significant correlations are observed only for age: the older the person, the higher the level of self-declaration of religious faith, although the correlation is weak. An increase in age by one year causes an increase in the level of self-declaration of religious faith by 0.018 points (± 0.005). At the same time, including the respondent’s age variable in the model does not change the strength of the relationship between the COVID-19 Disease Experience Scale, the Religious Meaning of the Pandemic Scale, and the self-declaration of religious faith. In turn, gender and the Sanitary Restrictions Scale do not create statistically significant relationships with the self-declaration of religious faith, and their inclusion in the model does not change the strength of the relationship of the remaining predictors with the explained variable.

The results are different for irregular practitioners (Table 4). Experiencing COVID-19 does not correlate with a self-declaration of religious faith. In other words, it does not matter if the people who describe themselves as irregular practitioners have had some experience with the disease, because they do not differ in their self-declaration of religious faith. The analysis of the scale describing the assignment of religious meaning to the pandemic leads to different conclusions. Here, the same correlation is noted as for regular practitioners: the more religious significance assigned to the pandemic, the higher the expected level of self-declaration of religious faith. An increase of one point on the Religious Meaning of the Pandemic Scale causes an increase of 0.890 points (± 0.048) on the Self-Declaration of Religious Faith Scale. It is worth emphasising that although the direction of the relationship between these variables is the same as in the case of regular practitioners, the strength of this relationship is slightly weaker.

**Table 4:** Results of the stepwise linear regression analysis for the subsample of irregular practitioners.

	B	Std. error	ß	T	*p*	Collinearity	
						Tol.	VIF
Step 1							
Constant	12.938	0.251		51.462	< 0.001		
Illness experience	−0.092	0.065	−0.062	−1.430	0.153	1.000	1.000
Step 2							
Constant	9.407	0.270		34.850	< 0.001		
Illness experience	−0.029	0.050	−0.020	−0.584	0.559	0.996	1.004
Pandemic’s religious significance	0.905	0.048	0.636	18.850	< 0.001	0.996	1.004
Step 3							
Constant	8.862	0.354		25.048	< 0.001		
Illness experience	−0.024	0.050	−0.016	−0.477	0.633	0.993	1.007
Pandemic’s religious significance	0.906	0.048	0.636	18.955	< 0.001	0.995	1.005
Age	0.011	0.005	0.079	2.368	0.018	0.998	1.002
Step 4							
Constant	8.812	0.427		20.641	< 0.001		
Illness experience	−0.024	0.050	−0.016	−0.475	0.635	0.993	1.007
Pandemic’s religious significance	0.907	0.048	0.636	18.937	< 0.001	0.995	1.005
Age	0.011	0.005	0.080	2.370	0.018	0.997	1.003
Gender	0.033	0.157	0.007	0.210	0.834	0.999	1.001
Step 5							
Constant	9.526	0.528		18.024	< 0.001		
Illness experience	−0.012	0.050	−0.008	−0.237	0.813	0.983	1.018
Pandemic’s religious significance	0.890	0.048	0.625	18.447	< 0.001	0.972	1.029
Age	0.012	0.005	0.089	2.649	0.008	0.981	1.019
Gender	0.046	0.157	0.010	0.294	0.769	0.998	1.002
Sanitary restrictions	−0.081	0.035	−0.078	−2.274	0.023	0.949	1.054

*Source: Authors’ research*; Step 1: ΔR² = .002, *p* = .153; Std. error = 2.32858; Step 2: ΔR² = .404, *p* < .001; Std. error = 1.79995; Step 3: ΔR² = .409, *p* < .001; Std. error = 1.79210; Step 4: ΔR² = .408, *p* < .001; Std. error = 1.79374; Step 5: ΔR² = .412, *p* < .001; Std. error = 1.78663

For irregular practitioners, there is an additional relationship that did not occur previously. The stronger their acceptance of sanitary restrictions in places of religious worship (understood as the need to limit access to these places during pandemics and the primacy of state authorities over church authorities in making decisions on restricting access), the lower their self-declared level of religious faith. An increase in acceptance of sanitary restrictions by one point on a 13-point scale is associated with a decrease of 0.081 points (± 0.035) on the Self-Declaration of Religious Faith Scale. Although the prediction is weak, the correlation between both variables is statistically significant.

The remaining results are identical to the results describing the subsample of regular practitioners. The greater the age, the stronger the self-declaration of religious faith: increasing the age by one year increases the score on the Self-Declaration of Religious Faith Scale by 0.012 points (± 0.005). Including one’s age, however, does not cause any changes in the model. The extent of experiencing COVID-19 still does not differentiate the self-declaration of religious faith, and the strength of the relationship between assigning religious meaning to the pandemic and the self-declaration of religious faith remains unchanged. Gender again does not show any statistically significant relationship with the self-declaration of religious faith and does not affect this type of relationship with other variables in the model.

The results of the stepwise linear regression analysis for the subsample of non-practising believers led to partially different conclusions compared with the previous models (Table 5). As with irregular practitioners, these results also do not indicate a statistically significant relationship between experiencing COVID-19 and a self-declaration of religious faith. Regarding the last variable, as in the previous models, the results indicate a statistically significant relationship with assigning religious significance to the pandemic. An increase of one point on the Religious Meaning of the Pandemic Scale coincides with an increase of 0.775 points (± 0.056) on the Self-Declaration of Religious Faith Scale.

**Table 5:** Results of the stepwise linear regression analysis for the subsample of non-practising believers.

	B	Std. error	ß	t	*p*	Collinearity	
						Tol.	VIF
Step 1							
Constant	10.930	0.253		43.250	< 0.001		
Illness experience	0.021	0.065	0.015	0.326	0.745	1.000	1.000
Step 2							
Constant	8.475	0.258		32.787	< 0.001		
Illness experience	−0.007	0.052	−0.005	−0.128	0.899	0.999	1.001
Pandemic’s religious significance	0.834	0.054	0.589	15.523	< 0.001	0.999	1.001
Step 3							
Constant	8.267	0.346		23.895	< 0.001		
Illness experience	−0.011	0.053	−0.008	−0.214	0.831	0.990	1.010
Pandemic’s religious significance	0.838	0.054	0.592	15.542	< 0.001	0.993	1.008
Age	0.004	0.005	0.035	0.905	0.366	0.985	1.015
Step 4							
Constant	8.241	0.428		19.256	< 0.001		
Illness experience	−0.011	0.053	−0.008	−0.217	0.828	0.988	1.012
Pandemic’s religious significance	0.838	0.054	0.592	15.523	< 0.001	0.992	1.008
Age	0.004	0.005	0.035	0.909	0.364	0.981	1.019
Gender	0.017	0.166	0.004	0.103	0.918	0.994	1.006
Step 5							
Constant	9.546	0.546		17.475	< 0.001		
Illness experience	0.011	0.052	0.008	0.208	0.835	0.976	1.025
Pandemic’s religious significance	0.775	0.056	0.548	13.920	< 0.001	0.905	1.105
Age	0.008	0.005	0.059	1.540	0.124	0.953	1.049
Gender	−0.010	0.164	−0.002	−0.058	0.953	0.993	1.008
Sanitary restrictions	−0.120	0.032	−0.151	−3.760	< 0.001	0.868	1.153

*Source: Authors’ research*; Step 1: ΔR² = .002, *p* = .745; Std. error = 2.17891; Step 2: ΔR² = .344, *p* < .001; Std. error = 1.76290; Step 3: ΔR² = .344, *p* < .001; Std. error = 1.76325; Step 4: ΔR² = .342, *p* < .001; Std. error = 1.76518; Step 5: ΔR² = .361, *p* < .001; Std. error = 1.74006.

However, unlike in the case of regular or irregular practitioners, age does not statistically significantly differentiate the self-declaration of religious faith of non-practising believers. Including this variable also does not change the strength of the relationship between the variable describing the degree of assigning religious meaning to the pandemic and the level of self-declaration of religious faith. The same applies to the gender variable, which does not statistically significantly differentiate the level of self-declaration of religious faith, as in the previous models.

For non-practising believers, similarly to irregular practitioners, an additional relationship emerges that was not observed among regular practitioners. The stronger their acceptance of sanitary restrictions in places of religious worship, the lower their self-declared level of religious faith. An increase in acceptance of sanitary restrictions by one point on a 13-point scale is associated with a decrease of 0.120 points (± 0.032) on the Self-Declaration of Religious Faith Scale. It is worth emphasizing that the prediction of self-declaration of religious faith by the acceptance of sanitary restrictions is stronger among non-practising believers than among irregular practitioners. This indicates a negative correlation between the acceptance of sanitary restrictions and the frequency of religious practices.

## Discussion

Constructed meanings based on theodicy references are used to encode the COVID-19 experience, and the more intense this experience, the more it triggered religious involvement. However, action programmes that can be implemented under the new conditions have not yet been developed based on these meanings, which would allow for overcoming the continuing contingency of religion, understood as an indeterminate risk, uncertainty, and randomness. The schematisation of religious meanings undergoes transformations caused by external factors, but it is difficult to define the direction of these changes in relation to human experiences and activities. The pandemic situation is characterised by the fact that official churches, as interpreted by Luhmann, have lost their already severely limited ability to link their decision-making processes to the relationships that members – who exhibit widely varying degrees of involvement – create between their religious and non-religious experiences and activities [22].

This article is an attempt to respond to the challenge posed in 2020 by Raewyn Connell: ”Quite simply, we need more imaginative social thinking, especially about the new structures of power, and new means of change and ways of organising. Not least, we need thinking about forms of social living that are practicable, humane, and capable of meeting future COVID-scale crises” [31]. The originality of the approach proposed in our study lies in pointing out the crisis of fixed communication systems caused by COVID-19. In order to examine it, the issues of religious practices in Poland were addressed, as their sanitary and political restrictions sparked controversy. The institutionally fixed sphere of symbolic reference to reality was disrupted, revealing the paradoxes of modern rationality. This phenomenon was pointed out by Jefrey C. Alexander and Philip Smith: ”COVID-time has been suffused with mystery, superstition, and trauma; peopled with god-like heroes; generative of myth, new interpersonal rituals, but also iconic circulations of familiar imagery; and it has been haunted by a relentless search for both the blame and the salvation of charismatic authority” [32]. The presented findings are of a sociological nature, offering an alternative to the descriptions of posthumanist sociology [33], emphasizing the value of the systemic approach over anthropocentrism (psychologism) and sociocentrism. Our findings complement research on behaviour during the COVID-19 pandemic dependent on trust in science [34], trust in government [35], or vulnerability to political influences [36]. The research, of course, forms part of the extensive body of analyses on the relationship between religion, religiosity, religious practices and the experience of COVID-19, especially in terms of assigning meaning to it and the conflict of meanings institutionally shaped within prevailing narratives.

The results of the stepwise linear regression analysis indicate that, regardless of the scope of religious practice, assigning religious meaning to the pandemic coexists with an increased level of the self-declaration of religious faith. The more religious meaning is attributed to the pandemic, the higher the expected level of the self-declaration of religious faith. Experiencing COVID-19 is relevant to the self-declaration of religious faith only among regularly practising believers.

There is a correlation here: the level of self-declaration of religious faith decreases with the greater severity of one’s experience with the disease caused by COVID-19 among representatives of this category. This is puzzling, as, on the one hand, one would expect that the importance of religious faith would increase when greater religious meaning is attributed to the pandemic. On the other hand, it is surprising that believers and practitioners regularly assign less importance to religious belief in their declarations when they have experienced the consequences of COVID-19 more severely. This correlation does not appear at all in the case of people who practise irregularly or who do not practise.

This may be explained by the fact that the interpenetration of the mental and religious systems becomes disrupted because the medical system, which gives the infection a sanitary meaning, treats infected persons in a specific way. Human beings – or rather, individual mental systems – did not come into direct contact with the coronavirus itself. People experienced a disease whose significance and information about it were mediated by sanitary institutions, which confirmed or did not confirm an infection. Diseases caused by the virus are so unusual that it was the test result that served as confirmation, rather than any other form of diagnosis. Even the severe course of the disease is associated with the test result, which is linked to symptoms such as high temperature, cough, shortness of breath, or pneumonia.

The test in some way shaped awareness of the disease and its semantic implications. Therefore, the stronger the experience of infection, the stronger the reliance on medical arguments in the managing COVID-19 infection. This can result in a reduction of religious references when interpreting messages that shape expectations and behaviours during the epidemic. It is difficult to determine whether, under such conditions of globalised communication and media influence, it is possible to reconcile the interpretation of the pandemic through the prism of religion with accepting the microbiological interpretation of COVID-19. Such attitudes were not alien to other epidemics in modern times, such as the Spanish flu in South Africa in 1918, when many religious leaders easily came to terms with modern medicine, believing that it merely described the conditions established by the Creator [3].

The surprising effect of this decline in the level of self-declaration of religious faith with the more severe experience of COVID-19 among regularly practising believers may stem from meanings constructed within the institutional Church. This meaning is expressed through the division between deeply religious people, who entrust themselves to the care of the Most High God, and those who have lost faith in the power of such protection against the coronavirus disease. The priority given to this supernatural protection over the sanitary restrictions and vaccinations contrasts with signs of weak faith in recognising the protection offered by the political system. The observed trend of the coexistence of a lower level of religious belief with more severe COVID-19 disease experiences among regular practitioners may be explained by cognitive dissonance [37] between meanings formed before and after experiencing the severe consequences of the disease. One possible way for these respondents to resolve this dissonance is to adapt to the requirements of the environment by lowering the level of declared importance of religious meanings.

The degree of acceptance of sanitary restrictions in places of worship plays a certain role in differentiating the level of self-declaration of religious faith primarily among non-practising believers. In their case, a declining level of self-declaration of religious faith can be observed alongside an attitude that accepts limiting the functioning of places of religious worship to mitigate the consequences of a pandemic and recognises the primacy of state authorities over entities managing places of worship in making such decisions. Their lack of personal involvement in practising religion would lead to the expectation that no such correlation would be observed. Before the pandemic, in Polish conditions, the prevailing programme was undertaking actions (according to Luhmann’s idea) that integrated religion with participation in religious rituals, especially Sunday Mass.

Religious rituals are a form of communication between the religious system and other social systems that create events for celebration readable by individuals. They derive meaning from it for their activities, different from their usual leisure activities, even when they do not participate in these rites. The sanitary restrictions have disrupted this communication system and necessitated the search for new references and the construction of a horizon of further possibilities. In the communication processes of individual systems, claims have emerged that define the logical boundaries of these systems within their environment.

Demographic variables such as gender and age do not play a significant role in differentiating one’s self-declaration of religious faith. However, as expected, the level of self-declaration of religious faith increases with age, and the strength of the relationship between these variables is weak and applies only to practising believers. On the other hand, gender does not show any statistically significant relationship with the level of self-declaration of religious faith in any of the subgroups studied. Thus, the H1 hypothesis was only partially confirmed. Experiencing COVID-19 may be such a dominant variable affecting religious behaviour that demographic variables, which under normal conditions differentiate the population, are statistically insignificant under pandemic conditions.

The interpenetration of the mental and social systems takes place at a level that goes beyond demographic differences. This is because it concerns the fundamentals of communication and the collision of the existing ways of coding events with the environment. At the level of basic experiences (the theodicy perspective), it introduces serious disturbances in transmitting information and expecting a positive result. Therefore, we can talk about the signs of the collapse of the world as we know it, meaning the disappearance of the clear boundary of meanings that guarantee the unity of the difference between given systems and their environments. It can thus be assumed that such events, taking place at the level of one’s basic experience of meaning, may, as happened in the past, cause crises, destruction, the annihilation of some epochs, and the beginning of others, while contributing to serious spiritual and intellectual changes [38].

Niklas Luhmann’s systems theory provides a useful lens for analyzing and interpreting the complex relationships and reciprocal irritations between the psychic system (individual consciousness) and the religious system in the context of the COVID-19 pandemic. In a functionally differentiated modern society, no functional system (including religion) can directly determine or fully control psychic systems. Instead, they can only irritate them, and religious communication produces meaning that may or may not be taken up by individual consciousness as relevant, anxiety-reducing, identity-affirming, or identity-threatening. The precise shape of this irritation depends on the contingency-management style of the religious code and on how tightly or loosely religion is coupled to other systems that were dominant during the crisis, especially the health system and politics. Poland represents a particularly distinctive configuration, not a universal template, and in other societal configurations, the dynamics of irritation look markedly different. Thus, the Polish case illustrates one specific historical constellation and cannot serve as a universal model. Luhmann’s theory anticipates precisely such diversity, and the same abstract mechanism (irritation via structural coupling) produces highly context-dependent empirical patterns whenever the structural and semantic conditions differ. The pandemic did not create a single global ”religious–psychic” dynamic but it exposed and amplified the existing, societally specific forms of coupling and contingency processing.

## Study Limitations

It is important to acknowledge three limitations of the present study. First, the authors relied on the respondents’ answers to a questionnaire, not on behaviour actually observed in the field, such as an observable increase or decrease in the frequency of various religious practices. Therefore respondents’ answers (or declarations) may be subject to recall error, measurement error, and social desirability bias, particularly among older respondents. Social desirability bias is especially evident in social science research when the questionnaire is thematically connected to a socially sensitive subject, such as religion. The greater likelihood of social desirability bias in older generations (as opposed to younger people) is due to different life values adhered to by these subgroups, with older generations appearing more susceptible to social control [39,40]. The second limitation is that the study is not longitudinal; therefore, conclusions can be drawn about the coexistence of the analysed variables, but not about causal relationships. The third limitation is that the research was conducted in a country with an established Catholic culture, so caution in the interpretation and generalization of the findings is recommended. However, this aspect of the study presents the promising prospect of conducting future comparative studies involving respondents from other religious contexts.

## Conclusions

The findings of this study demonstrate that the COVID-19 pandemic was significantly correlated with both the intensity and character of religious attitudes among Polish believers. The interpenetration of mental and religious systems, as articulated through Luhmann’s social systems theory, contributed to the emergence of new meanings and modes of communication, particularly under conditions of crisis and uncertainty. Our empirical analysis, based on a statistically representative sample, indicates that the pandemic-related experiences were a key predictor of religious behaviour, reducing the explanatory power of traditional demographic variables. These results highlight the importance of incorporating theodicy-related perspectives when interpreting psychological and spiritual responses to large-scale societal disruptions. For readers, this underscores the need to address both mental health and religious dimensions in future research and intervention strategies, especially during periods of widespread adversity. The study’s implications are also relevant for policymakers, educators, and religious leaders, who should recognise that crisis conditions may substantially reshape the foundations of social communication and belief systems. By elucidating the interpenetration of mental and religious processes, we propose a framework for navigating future crises through a holistic, systems-oriented approach.

In this context, it seems that in situations of widespread threats – such as pandemics, wars, social unrest, or economic crises – closer cooperation between the state and religious institutions should be considered. Such cooperation could, on the one hand, ensure relatively safe access to places of worship, which may provide not only spiritual support but also psychological relief by helping to reduce anxiety and stress levels. On the other hand, it could support public health efforts by counteracting attitudes that discourage the use of evidence-based protective measures in favour of exclusive reliance on divine protection. At the micro-level, therapists, psychologists, clergy, and social workers should recognize, when interacting with their clients/patients, the extent to which these individuals attribute religious meaning to negative life events that motivate them to seek help. Based on our findings, it is expected that among believers such religious interpretations of adverse experiences may correlate with an increased importance of religious faith. At least for believers, this may be a mechanism for reducing anxiety and stress levels and for enhancing subjective well-being.
